# Editorial: Polymeric nanoparticles for sustainable plant agriculture and food industry

**DOI:** 10.3389/fpls.2023.1183938

**Published:** 2023-04-12

**Authors:** Mariya Khodakovskaya, Marta Marmiroli

**Affiliations:** ^1^ Department of Biology, University of Arkansas at Little Rock, Little Rock, AR, United States; ^2^ Department of Chemistry, Life Science and Environmental Sustainability, University of Parma, Parma, Italy

**Keywords:** green nanoscience, natural nanopolymers, plant agriculture, sustainability, biocompatibility

Nanotechnology has great potential to provide new solutions to major challenges of plant agriculture including deep climate change, water shortages, and a growing global population that will reach 9.8 billion in 2050. Therefore, it is important to find food security solutions in the coming years. Nanotechnology applied to agriculture and food packaging can help in attaining these goals of reinforcing food security. Recent studies have offered insight into the synthesis of new polymeric nanomaterials that are cheap, biodegradable, safe, biocompatible, and easily reproducible from a natural source. Both synthetic and natural nanopolymers can be used as nano-delivery systems, sensors, materials for food packaging, and many other agricultural applications including protection against climatic events such as frost damage. Although naturally derived particles can be degraded in the environment with time, synthetic polymers and nanoparticles are still raising concerns in the population related to possible environmental leaching and consequent contamination. This Research Topic has combined three mini-reviews and one research paper describing new developments involving the understanding of interactions between plants and biodegradable polymeric materials derived from natural sources. The authors reported the most recent applications of such nanomaterials for the improvement of plant agriculture, the food industry, and plant genetic engineering.

A mini review by Vinzant et al. summarized the recent information about the most promising classes of polymeric nanomaterials derived from various natural sources and discussed their potential use for the optimization and enhancement of agricultural practices. In an effort to keep things sustainable and cost-effective, it is best to derive nanoparticles from waste products and renewable materials. Additional consideration should be given to dietary preference and restriction, which gives some selection against animal-derived proteins for applications regarding food crops. Therefore, the focus of their research encompasses several polymeric nanomaterials that are important in terms of affordability, biocompatibility, and efficiency as nanoparticles. Thus, the use of promising natural nanopolymers such as nanocellulose, lignin, chitosan, zein, arabinoxylan, and DNA nanostructures was discussed towards their applications for seed coatings, soil supplementation, post-harvest packaging, delivery of insecticides and nucleic acids. DNA origami has gained some recent attention as a potential method of delivering genetic material into eukaryotic cells. DNA origami takes advantage of the classic Watson-Crick base pairing to design complex 3D shapes with DNA. These DNA nanoforms are designed using computer software such as caDNAno or Tiamat, and have been used to engineer molecular machines, which can utilize input logic gates based on aptamer conformation or be used as vessels for biomolecule delivery.

The mini-review by Gigli et al. is focused on new applications of lignin-based nanomaterials. Special attention was paid to the use of lignin-based nanoparticles as nanocarriers for the controlled delivery of fertilizers, pesticides, and herbicides. In this review, the authors present and discuss the current advancements in the preparation of lignin nanoparticles for the controlled release of pesticides, herbicides, and fertilizers, as well as the latest findings in terms of plant response to their application. Within this mini-review, special attention has been paid to the state-of-the-art literature concerning the release performance of the lignin-based nanomaterials, whose efficiency is compared with the conventional approaches. The traditional methods for crop protection require repeated applications of large volumes of active species at high initial dosages. Moreover, the non-controlled delivery causes a time limited biocidal protection and causes the ubiquitous presence of biocides in the environment, resulting in biocide resistance and soil/water/food chain contamination. In this context, lignin represents a green matrix for the design of sustainable biocide delivery systems, which are an effective tool for a controlled-release and stimuli-responsive delivery. Finally, the major challenges and the future scenarios of lignin-based nano-enabled agriculture are considered in the field of agriculture and food security.

The research paper of Arnoldussen et al. highlighted applications of nanocellulose, a relatively recently developed nano-sized material with great potential for agricultural needs. The authors described the innovative use of cellulose nanocrystals for the creation of an insulating film around buds for increasing the cold tolerance of crops. Cold damage has caused more economic losses to fruit crop growers in the U.S. than any other weather hazard, making it a perennial concern for producers. Cellulose nanocrystals (CNCs) represent a new generation of renewable bio-nanomaterials, with many unique physical and chemical properties, including their low thermal conductivity. Our team has developed a process for creating CNC dispersions that can be sprayed onto woody perennial crops, forming a thin insulating film around buds which has been shown to increase cold tolerance. Cellulose nanocrystals are bionanomaterials composed of uniform crystalline structures of cellulose with a length of 100 to 300 nm and a width between 5 and 15 nm. These nanocrystals, the authors said, can be renewably produced on an industrial scale from different plant materials such as wood pulp and agricultural byproducts (e.g., soybean, corn, sugarcane bagasse, hemp). The unique properties of nanocellulose have led to its use in many industrial sectors, not only agriculture, including paints and coating, polymer composites, catalysis, cosmetics, biosensors, drug delivery, and medical devices. The authors saw that lethal freezing temperature significantly lowered using digital scanning calorimetry (DSC). The DSC also showed that the energy released during the exotherms increased with treatment and time, suggesting an additional mechanism involving water relations within the treated buds. This increase in water could be related to the ability of the coating to slow nucleation and increase the amount of supercooled water during freezing or from the coating’s ability to decrease evapotranspiration on apple and cherry plants.

Cao et al. summarized available information about novel applications of cellulose-based hydrogels derived from bamboo in food packaging and plant agriculture. They purport that researchers recently have extracted cellulose from bamboo and generated a variety of cellulose-based functional hydrogels with excellent properties by various cross-linking methods. In addition, a variety of multifunctional hybrid cellulose-based hydrogels have been constructed by introducing functional components or combining them with other functional materials, thus expanding the breadth and depth of their applications. In the minireview is summarized that nowadays, cellulose-based hydrogels have wide applications in food packaging, plant agriculture, environment, biomedicine, personal care products, and energy electronics due to their hydrophilicity, biodegradability, biocompatibility, non-toxicity, and remarkable solvent uptake. For example, cellulose-based hydrogels have been widely used in food packaging due to their excellent properties such as low cost, light weight, biodegradability, and good mechanical properties. In recent years, efforts have been made to explore alternatives to replace petroleum-based packaging materials to solve ecological problems, such as energy crisis and global warming. Another instance is the application in agriculture where hydrogels are receiving great attention since they are extremely hydrophilic polymers. For instance, hydrogels were prepared with polyacrylamide (PAAm), methyl cellulose (MC), and calcium montmorillonite (MMt). The produced hydrogels were utilized for the controlled release of fertilizers through the sorption and desorption studies of a nitrogenated fertilizer, urea [CO(NH2)2]. The prepared hydrogels show quite homogeneous foliaceous structures. The pore morphology of hydrogels did not change significantly with the addition of clay. However, the pore size increased after the hydrolysis treatment. As a result, hydrogels show the controlled release of urea in different pHs. The Authors say that it has been found that the addition of clay minerals improved the controlled release of urea when hydrogels are prepared from bamboo-based cellulose and other materials for seed culture applications.

Overall, this paper selection is a contribution to the growing area of “Green Nanoscience” by providing a summary of recent reports on the successful agricultural use of cost-efficient, biocompatible nano polymers derived from a sustainable source. This research can lay a good exploration foundation for the functionalization and value-additive applications of natural polymers such as cellulose and lignin from different sources to be utilized in the improving of agricultural fertilizers and pesticide delivery.

## Author contributions

All authors listed have made a substantial, direct, and intellectual contribution to the work and approved it for publication.

